# High Affinity Decynium-22 Binding to Brain Membrane Homogenates and Reduced Dorsal Camouflaging after Acute Exposure to it in Zebrafish

**DOI:** 10.3389/fphar.2022.841423

**Published:** 2022-06-09

**Authors:** Georgianna G. Gould, Priscilla A. Barba-Escobedo, Rebecca E. Horton, Lynette C. Daws

**Affiliations:** ^1^ Center for Biomedical Neuroscience, University of Texas Health Science Center at San Antonio, San Antonio, TX, United States; ^2^ Department of Cellular and Integrative Physiology, University of Texas Health Science Center at San Antonio, San Antonio, TX, United States; ^3^ Department of Endodontics, University of Texas Health Science Center at San Antonio, San Antonio, TX, United States; ^4^ Department of Pharmacology, University of Texas Health Science Center at San Antonio, San Antonio, TX, United States

**Keywords:** anxiety, black–white plus maze, *Danio rerio* (zebrafish), dive tank, SLC22A, pseudoisocyanine, uptake 2 transporters, predator avoidance

## Abstract

Organic cation transporters (OCTs) are expressed in the mammalian brain, kidney, liver, placenta, and intestines, where they facilitate the transport of cations and other substrates between extracellular fluids and cells. Despite increasing reliance on ectothermic vertebrates as alternative toxicology models, properties of their OCT homologs transporting many drugs and toxins remain poorly characterized. Recently, in zebrafish (*Danio rerio*), two proteins with functional similarities to human OCTs were shown to be highly expressed in the liver, kidney, eye, and brain. This study is the first to characterize *in vivo* uptake to the brain and the high-affinity brain membrane binding of the mammalian OCT blocker 1-1′-diethyl-2,2′cyanine iodide (decynium-22 or D-22) in zebrafish. Membrane saturation binding of [^3^H] D-22 in pooled zebrafish whole brain *versus* mouse hippocampal homogenates revealed a high-affinity binding site with a K_D_ of 5 ± 2.5 nM and Bmax of 1974 ± 410 fmol/mg protein in the zebrafish brain, and a K_D_ of 3.3 ± 2.3 and Bmax of 704 ± 182 fmol/mg protein in mouse hippocampus. The binding of [^3^H] D-22 to brain membrane homogenates was partially blocked by the neurotoxic cation 1-methyl-4-phenylpyridinium (MPP+), a known OCT substrate. To determine if D-22 bath exposures reach the brain, zebrafish were exposed to 25 nM [^3^H] D-22 for 10 min, and 736 ± 68 ng/g wet weight [^3^H] D-22 was bound. Acute behavioral effects of D-22 in zebrafish were characterized in two anxiety-relevant tests. In the first cohort of zebrafish, 12.5, 25, or 50 mg/L D-22 had no effect on their height in the dive tank or entries and time spent in white arms of a light/dark plus maze. By contrast, 25 mg/L buspirone increased zebrafish dive tank top-dwelling (*p* < 0.05), an anticipated anxiolytic effect. However, a second cohort of zebrafish treated with 50 mg/L D-22 made more white arm entries, and females spent more time in white than controls. Based on these findings, it appears that D-22 bath treatments reach the zebrafish brain and have partial anxiolytic properties, reducing anti-predator dorsal camouflaging, without increasing vertical exploration. High-affinity binding of [^3^H] D-22 in zebrafish brain and mouse brain was similar, with nanomolar affinity, possibly at conserved OCT site(s).

## Introduction

Organic cation transporters (OCTs) are transmembrane proteins of the solute carrier family SLC22A responsible for bi-directional facilitated sodium-independent electrogenic transport of compounds that are mono or multivalent cations at physiological pH. They are blocked by hydrophobic polyamines and steroid hormones (Koepsell et al., 2007; Hill et al., 2011; Sala-Rabanal et al., 2013). In mammals, there are three isoforms: OCT1 is richly expressed in the liver, kidneys, and gut; OCT2 is widely found in the brain, kidneys, and to a lesser extent, in other peripheral organs; and OCT3 is predominant in the heart, lungs, adipose tissue, placenta, and brain, and also occurs in other peripheral organs (Koepsell et al., 2007; Nies et al., 2011; Koepsell, 2020; Samodelov et al., 2020; Sweet, 2021). Substrates of mammalian OCTs include the biogenic amines serotonin, norepinephrine, dopamine, and histamine; antioxidants; vitamins such as choline and thiamine; metabolites such as guanidine or putrescine; xenobiotic compounds including drugs such as metformin; and cationic neurotoxins such as the 1-methyl-4-phenyl-1,2,3,6-tetrahydropyridine (MPTP) cation, 1-methyl-4-phenyl pyridinium (MPP+), or paraquat cations that are used to model Parkinson’s disease in rodents (Cui et al., 2009; Rappold et al., 2011; Nies et al., 2011; Bönisch, 2021; Yee and Giacomini, 2021). The pseudoisocyanine 1-1′-diethyl-2,2′cyanine iodide (decynium-22 or D-22) blocks human and mouse OCTs at low nM concentrations, and because of this property, D-22 is a useful pharmacological tool in the studies of OCT functions (Hayer et al., 1999; Hayer-Zillgen et al., 2002; Fraser-Spears et al., 2019; ; Bönisch, 2021).

Human gene polymorphisms affecting the expression or function of OCTs are associated with substance abuse (Aoyama et al., 2006; Bousman et al., 2009; Bergen et al., 2014), resistance to drugs such as metformin (Nies et al., 2009; Becker et al., 2010; Chen et al., 2010; Chen et al., 2015), and a more rapid progression of Parkinson’s disease (Becker et al., 2011). Developmental language delays, hypotonia, and motor speech disorders were evident in two cases of chromosome 6q deletions that impacted OCT2 and OCT3 genes (Peter et al., 2017). Given this, gene polymorphisms impacting OCT expression or function may contribute to psychiatric disorders such as depression, anxiety, and autism spectrum disorders, but testing this hypothesis requires further clinical investigations (Daws et al., 2013; Gasser and Daws, 2017; Daws, 2021). In humans and rodents, OCTs have lower affinity for monoamine transmitters than sodium-dependent serotonin (SLC6A4), norepinephrine (SLC6A2), or dopamine (SLC6A3) transporters (Fraser-Spears et al., 2019). SLC6A transporters are the primary mediators of monoamine clearance, so they are called “uptake 1,” while in most circumstances, OCTs play an auxiliary role in monoamine clearance and are referred to as “uptake 2” (Daws et al., 2013; Gasser, 2021). However, OCTs have a greater capacity to clear monoamines at high concentrations or if SLC6A transporters are compromised (Daws, 2009).

Rodent studies show that OCTs modulate monoamine availability in the brain to shape mood and behaviors. However, OCT effects on anxiety have been equivocal. For example, OCT3 knockout mice were more active and exhibited less anxiety in some tests (Wultsch et al., 2009), but were more stressed, anxious, and less sensitive to psychostimulants in others (Vialou et al., 2008). OCT3 knockout males had lower social interaction preferences than wild types (Garbarino et al., 2019), which may stem from early developmental dysregulation of serotonin neurotransmission (Karahoda et al., 2020). Systemic D-22 treatment blocked the serotonin uptake and produced antidepressant-like effects independent of the serotonin transporter (Baganz et al., 2008; Horton et al., 2013). Co-administration of D-22 with monoamine reuptake inhibitors enhanced antidepressant effects in wild-type mice (Krause-Heuer et al., 2017; Bowman et al., 2020). Since acute effects of D-22 on anxiety-relevant behaviors were not well characterized, one goal of this study was to use zebrafish to look for such effects. We used two different tests, dive tank and light–dark plus maze with established protocols to assess acute D-22 effects on anxiety-based behaviors (Gould, 2010a; Sackerman et al., 2010; Connors et al., 2014).

OCTs have similar structure and function in bacteria, plants, and animals (Koepsell et al., 2007). Two OCT orthologs were recently discovered and characterized in zebrafish (*Danio rerio*): drOCT1 on chromosome 20 and drOCT2 on chromosome 17; they are most syntenic with human OCT1, OCT2, and OCT3 which occur as a gene cluster on chromosome 6 (Mihaljevic et al., 2016). The expression of drOCT1 was high in liver and kidneys, while drOCT2 was high in eyes, and sex dependent expression in muscle, gonads, and gills. Brain drOCT expression in males was higher than that in females. Furthermore, the same group found many similarities in functional properties among human OCTs and zebrafish drOCT1 through homology modeling and transfection of drOCT1 into human embryonic kidney (HEK293T) cells for fluorescent substrate uptake saturation and concentration-dependent inhibition assays (Mihaljević et al., 2017). However, neither ligand-binding properties of D-22 in zebrafish nor if bath exposures to D-22 can even reach the brain were previously reported. Since pharmacological profile differences occur even among mouse, rat, and human OCTs for substrates and blockers (Maier et al., 2021), the second goal of this study was to characterize the high-affinity binding properties of radiolabeled D-22 in zebrafish whole-brain membrane homogenates that could underlie any acute changes in their anxiety behaviors.

## Materials and Methods

### Animals

All procedures involving live zebrafish were approved under protocol #090124 by the University of Texas Health Science Center at San Antonio Institutional Animal Care and Use Committee in accord with the National Institutes of Health guidelines (http://oacu.od.nih.gov/ARAC). A hundred zebrafish (*Danio rerio*) 4–6 months old with a mean ± SEM weight of 0.285 ± 0.016 g were obtained in 2009 from Aquatic Eco-Systems Inc (Apopka, FL, United States). Subsequently, 48 zebrafish weighing 0.171 ± 0.013 g were obtained for additional behavior tests from Carolina Biological Supply Co. (Burlington, NC, United States). Fish were housed in mixed-sex groups of six to eight for 2–3 months in the original 2009 studies, and one week for a more recent study to acclimate before use in 3 L tanks of a benchtop flow through aquatic habitat (Aquatic Eco-Systems, Apopka, FL, United States) filled with 25–27°C deionized water (Nanopure, Barstead, Dubuque, IA, United States) supplemented with 200 mg/L “Instant Ocean” salts (Aquarium Systems, Mentor, OH, United States), pH = 7.2–7.6. Light/dark cycles were 14:10 h (lights on at 700 h and off at 2,100 h). Fish were fed “Top Fin” tropical flakes once per day (Pacific Coast Distributing, Phoenix, AZ, United States).

Six constitutive serotonin transporter knockout heterozygous six-month-old male mice weighing 26 ± 2 g came from an in-house breeding colony established by the founders generously provided by Dr. Dennis Murphy (NIMH) in 1999 (Bengel et al., 1998). The mice on a congenic C57BL/6J background were littermates derived from heterozygous mating and were raised and housed together in same-sex groups from weaning until use in radioligand binding experiments. The mice were housed in a temperature- and humidity-controlled vivarium under a 12-h light/dark cycle (lights on at 600 h) with *ad libitum* access to rodent diet (Teklad 7,912 irradiated, Envigo) and water (reverse osmosis, acidified to pH 2.5–3 with HCl). Their use for tissue was IACUC-approved under protocol #020014.

### Acute Drug Treatments of Zebrafish for Behavior Testing

Initially, 45 zebrafish, a mixed assortment of males and females, were randomly assigned to seven different compound exposure groups. All compounds were from Sigma Aldrich (St. Louis, MO, United States). Fish were individually bath-exposed for 5 min to drugs dissolved in 20 ml solutions in 50-ml glass beakers. Dimethyl sulfoxide (1%) was used as a control or vehicle for all treatments. The anti-anxiety drug buspirone, a serotonin 5-HT_1A_ receptor partial agonist, was used at 25 mg/L as a positive control since it had anxiolytic effects in zebrafish dive tank tests (Bencan et al., 2009). D-22 was tested at 12.5, 25, and 50 mg/L. In addition, the mammalian stress hormone corticosterone (CORT) was administered at 25 mg/L. This corticosterone treatment was included because in vitro it has been shown to block mammalian OCTs (Gasser, 2021). Red food dye (five drops, red #40 and #3, H.E.B, San Antonio, TX, United States) was used as a color control for the D-22 (50 mg/L) solution since it had the same wavelength and similar amplitude in the spectrophotometer (DU-600, Beckman, Brea, CA, United States) absorption spectrum.

In a follow-up experiment, 24 females and males were used. Sex of these fish was visually determined by a combination of early morning inspection for female genital papilla as described by Yossa et al. (2013) and male yellow or golden-colored pectoral fin breeding tubercles as described by McMillan et al. (2015) before assigning D-22 (50 mg/L) or vehicle treatments to 12 of each sex. These characteristics are consistent with descriptive colorations used to determine sex in the initial study as per Paull et al. (2008). After 5 min bath exposures to treatments or controls for all experiments, zebrafish were placed in 100 ml habitat water for 5 min to rinse and were given drugs to approach maximal physiological efficacy before a series of two anxiety-relevant tests of response to a novel environment.

### Novel Environment Test 1: Height in Dive Tank Water Column

A 4-L triangular acrylic tank (Aquascene 1, TopFin, Phoenix, AZ) was filled 18 cm deep with 3.5 L of home tank water. Lines dividing it into thirds were drawn in advance on the outside with a permanent marker. The tank sat on a black countertop, with a 24 cm × 22 cm whiteboard against its back wall to enhance contrast. After drug exposure and 5 min in holding, each zebrafish was placed in a dive tank, observed, and digitally recorded (HP Photosmart R742, OfficeMax, United States) for 5 min to measure the time spent in the top 2/3 *vs*. the bottom 1/3 of the tank, as previously described by Sackerman et al. (2010). In the initial study, Bartlett’s test (*p* = 0.7) showed standard deviations among groups that were similar so the group means were compared by the one-way analysis of variance (ANOVA) with Dunnett’s multiple comparisons used for *post hoc* analysis of significant effects. In the follow-up study, a two-way (sex × treatment) ANOVA with Sidak’s *post hoc* analysis for significant outcomes was performed using GraphPad Prism nine.

### Novel Environment Test 2: Light-Dark Plus Maze Arm Preference

**FIGURE 1:**
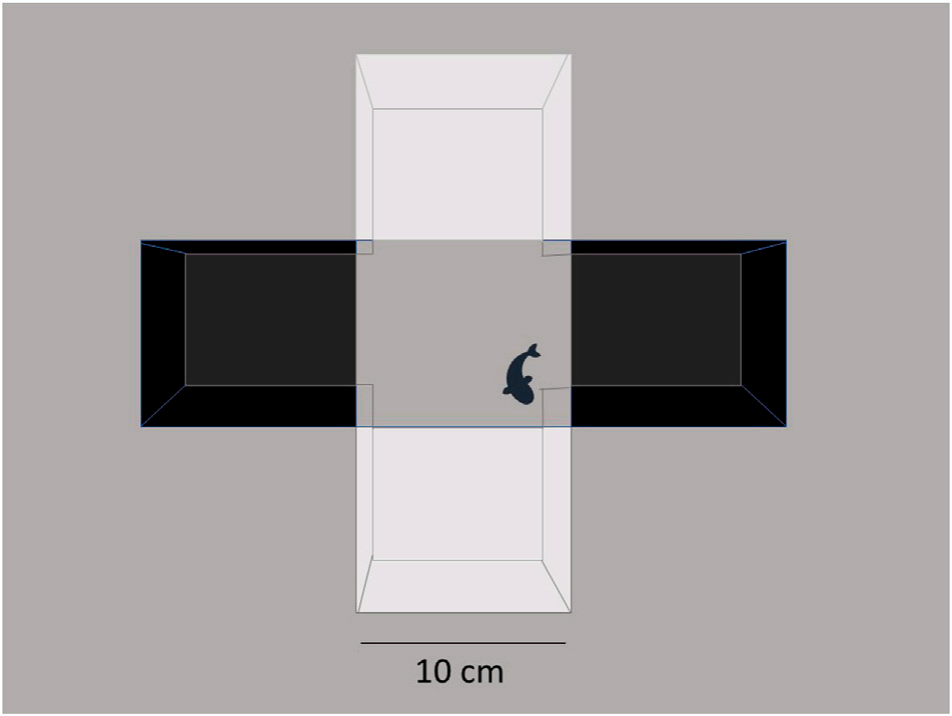
Light/dark plus maze. The aquatic cross maze is set up with a 3 × 3 box configuration at the intersection of the maze. Fish explore the environment under bright light (800 lumens from a 60 W bulb), and the behavior is video-recorded for 5 min. Treatment-blind observers collect data on time spent in different areas of the maze.

After dive tank tests, fish were tested in the aquatic light–dark plus maze that was performed as described by Gould (2010a). Briefly, a clear acrylic cross maze (Noldus, Leesburg, VA, United States) was used in a 30 cm^2^ × 30 cm^2^ plus configuration. Opposite arms were covered with black polyethylene and the other two with white polyethylene 10 cm^2^ squares (Figure 1). The gray background of the copy stand (Kaiser RS1, B&H Photo, New York, NY) showed through the middle 10 cm^2^ section of the maze. The maze was filled to a depth of 4 cm. A 60-W incandescent desk lamp (800 lumens) was mounted on the copy stand behind the digital camera (HP Photosmart R742) above the maze for testing. Each zebrafish was placed in the center of the maze to start, and the total number of line crosses, the percent of the total entries into the white arms, and the total time spent in the white arms were observed from 5-min video recordings. For the first experiment, Bartlett’s test was performed to determine if standard deviations differed among groups, and if they did not, one-way ANOVA was performed, but if they did, then Welch’s ANOVA was performed, with Dunnett’s multiple comparisons for the *post hoc* analysis of significant effects. For the follow-up experiment, two-way (sex × treatment) ANOVA was performed with Sidak’s *post hoc* test for significant findings using Prism nine. After behavior tests, each fish was weighed and euthanized by submersion in ice water for 5 min and subsequent decapitation to effect.

### Uptake of [^3^H] D-22 from Bath Water Into Zebrafish Muscle, Viscera, and Brain

A total of six adult zebrafish, three males and females each, were individually exposed to 25 nM [^3^H] D-22 (25 Ci/mmol, ARC, Boston, MA (11.4 μg/L)) in 25 ml habitat water in a 50-ml beaker for 10 min. Fish were removed from the radioligand bath with forceps, anesthetized and rinsed with a 30 s dip in ice water, and euthanized by decapitation on an ice-chilled glass Petri dish with a scalpel. From each fish, [^3^H] D-22 labeled zebrafish brain, visceral organs (heart, gastrointestinal tract, gall bladder, spleen, and liver), and a segment of the skinned lateral muscle were removed as described by Gupta and Mullins (2010), and then weighed and placed in 1.5 ml microcentrifuge tubes containing 200 ml scintillation cocktail (Ecolume, Fisher Scientific, United States). Labeled brains and muscles were mechanically homogenized with a teflon pestle and transferred to 8 ml scintillation vials (Beckman Mini Poly-Q, Fisher Scientific, United States), to which 4 ml of scintillation cocktail was added. Tissue homogenates in vials were vortexed, then mixed on an orbital shaker overnight. The [^3^H] label (DPM) was measured on a Packard 1900 TR liquid scintillation counter (Packard Instrument Co., Downers Grove, IL) with efficiency of 40%.

### Saturation Binding of [^3^H] D-22 in Zebrafish and Serotonin Transporter-Deficient Mice

Saturation assays were performed to determine the specific binding of D-22 to uptake 2 sites in mouse hippocampus and zebrafish whole brain. Heterozygous serotonin transporter knockout mice were used because their hippocampal OCT3 expression was upregulated *vs*. the wild-type mice (Baganz et al., 2008). A 50 mM Tris HCl, 120 mM NaCl, 5 mM KCl buffer, pH 7.4 at 25°C was used. Hippocampi from two mice were combined to produce one membrane homogenate, and 12 zebrafish whole brains from males and females were combined to produce the other membrane homogenate in 25 ml buffer each. Both were homogenized separately at 26,000 rpm for 1 min with a Polytron tissue homogenizer (Brinkman Instruments, Westbury, NY, United States). Homogenates were centrifuged for 10 min at 36,000 × g at 4°C using a JA 25.50 rotor (Avanti J-E, Beckman Coulter, Indianapolis, IN, United States). The supernatant was discarded, and the pellet was resuspended in 25 ml buffer on ice using a Potter Elvehjem 10-ml glass and teflon homogenizer. The homogenate was centrifuged again for 10 min at 36,000 × g. These final pellets were resuspended to obtain an approximate protein concentration of 1 mg/ml, determined using the Bradford reagent (Sigma, St. Louis, MO), and colorimetric detection was performed at 595 nm using a plate reader (Spectra Max 190, Molecular Devices, San Jose, CA, United States).

For the incubations, a 100-µL homogenate was added to a tube containing 150 µL buffer with [^3^H] D-22 at eight concentrations ranging from 0.1 to 14 nM [^3^H] D-22 since concentrations exceeding this range are not pharmacologically relevant (Horton et al., 2013). To block any potential D-22 binding to uptake 1 transporters in mice or fish (Gould et al., 2007), 25 nM each of sertraline and mazindol were added to the buffer. To define the non-specific binding of [^3^H] D-22, 25 µM MPP+ and 25 µM cold D-22 were added to the second and third groups of tubes, respectively. Each condition was reproduced in duplicate tubes. The solutions were incubated at room temperature on an orbital mixer for 60 min, and incubation was terminated by the addition of 4 ml of buffer, pH 7.4 at 4°C. Labeled homogenates were captured by filtration under vacuum on glass fiber filters pre-soaked in 0.5% polyethyleneimine (Sigma) with a Brandel tissue harvester (Gaithersburg, MD). Filters were washed twice more with 4 ml of buffer. Radioactivity trapped by the filters was measured on a scintillation counter (Packard Instrument Co., Downers Grove, IL) with 40% efficiency. Non-linear and linear curve fits were performed with Delta Graph (V5, Red Rock Software, Salt Lake City, UT, United States) and confirmed using Prism (V5 for Mac OS 10, Graph Pad, LaJolla, CA, United States).

## Results

### Zebrafish Anxiety-Relevant Behaviors in Novel Environments After Acute D-22 Treatment: Initial Study

**FIGURE 2:**
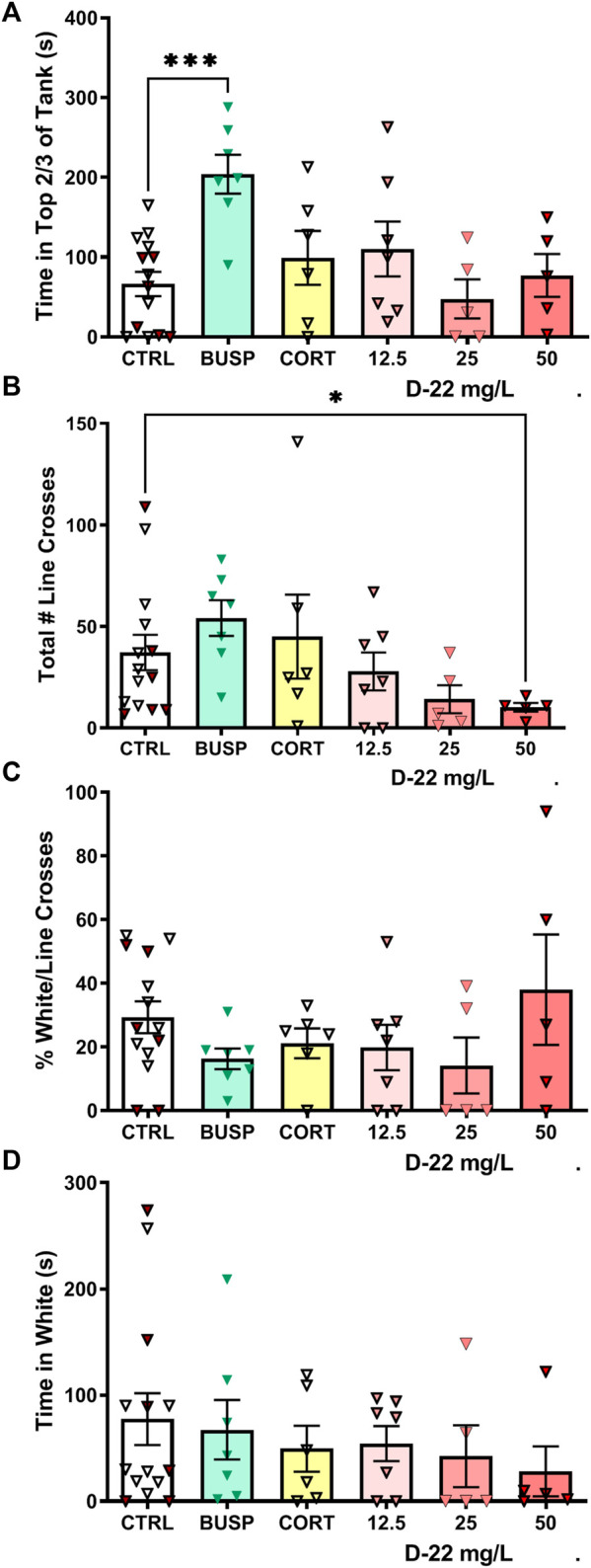
Acute effects of D-22 on behaviors in novel environment-based anxiety tests. A total of 45 zebrafish were tested with sample sizes ranging from 5 to 15 fish with both males and females in every group. **(A)** Only the anti-anxiety drug buspirone increased the amount of time zebrafish spent at the top of the dive tank. **(B)** D-22 at 50 mg/L reduced the number of line crosses made by zebrafish exploring the light/dark plus maze. **(C)** There were no effects of D-22 or any other treatment on the percentage of white box entries/total entries. **(D)** There was no effect of D-22 or any other treatment on the time spent in white boxes. In all figures, for controls (CTRL), the filled symbols are the food color controls and open symbols are uncolored control solutions. CORT = corticosterone at 25 mg/L. Mean and S.E.M. are shown.

**Dive Tank:** Initially, the behavior of zebrafish exposed to red food dye matching the wavelength with a similar intensity to the 50 mg/L D-22 solution was compared with habitat water controls. There was no difference in top dwelling between these two groups [t (13) = 1.221, *p* = 0.24], so the data were pooled into a single control group of 15 fish. The time in the top 2/3 of the dive tank for this control group was compared with the average times for five to seven fish treated with buspirone, corticosterone, or three concentrations of D-22 treatments. Only the zebrafish treated with 25 mg/L buspirone spent more time in the top 2/3 of the tank [F (5, 39) = 4.786, *p* = 0.0017, Dunnett’s *p* = 0.0004], as shown in Figure 2A.

**Light–Dark Plus Maze:** The red food dye-control group did not differ from the habitat water-control group in number of line crosses into different boxes [t (13) = 0.1836, *p* = 0.86], percentage white of total line crosses [t (13) = 0.9615, *p* = 0.3538], and time in white arms [t (13) = 0.4692, *p* = 0.6467], so these control groups were pooled. The standard deviations for line crosses differed among the groups (Bartlett’s *p* = 0.004), so Welch’s ANOVA was used. The total number of line crosses (entries into center, white, or black boxes) in the light–dark maze was reduced only in the 50 mg/L D-22 treatment group [W (5.000, 14.49) = 6.130, *p* = 0.003, Dunnett’s *p* = 0.04], as shown in Figure 2B. The percentage of white/total line crosses also had different standard deviations among groups (Bartlett’s *p* = 0.03), but the means did not differ between treatments [W (5.000, 14.11) = 1.288, *p* = 0.323], as shown in Figure 2C. For time spent in white boxes, the standard deviations among groups were similar (Bartlett’s *p* = 0.4657), and there were no significant differences among groups [F (5, 39) = 0.4940, *p* = 0.7787], as shown in Figure 2D.

### Follow-Up Study: Acute D-22 50 mg/L Treatment in Females *Versus* Males

**FIGURE 3:**
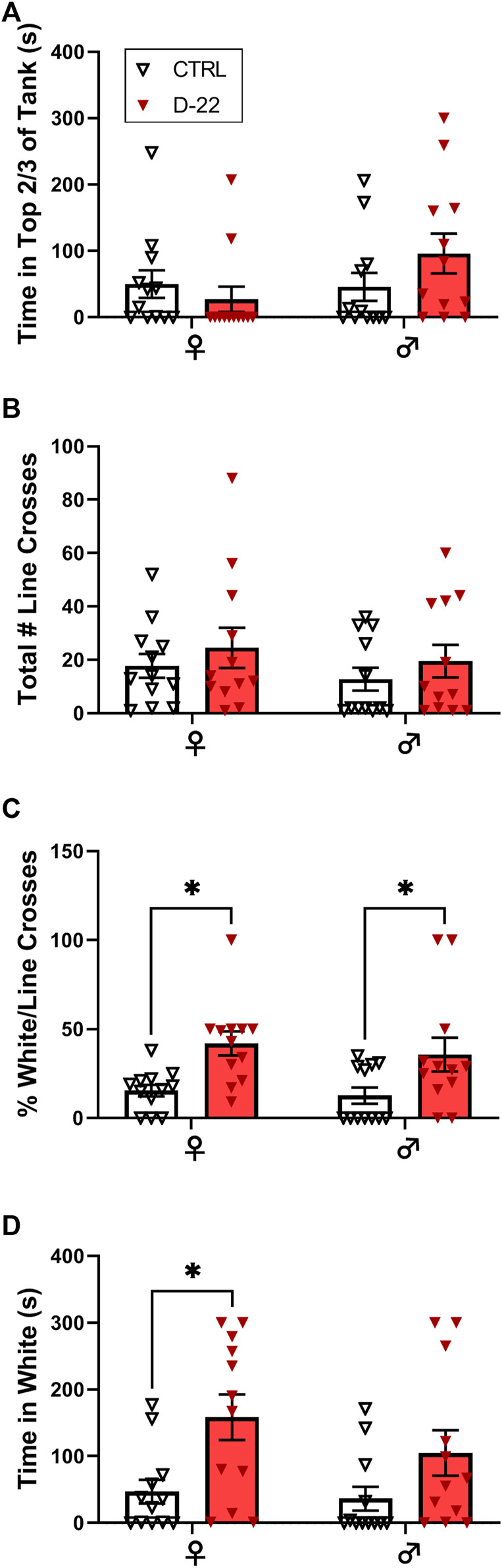
Acute effects of D-22 (50 mg/L) on female *versus* male zebrafish in anxiety tests. A total of 48 zebrafish, 12 each of males and females, were treated with D-22 or a vehicle control for 5 min, washed for 5 min, and tested in a battery of anxiety-relevant behaviors. **(A)** There was no effect of D-22 treatment or sex on top dwelling in the dive tank. **(B)** There was no effect of D-22 treatment or sex on exploration of the maze as measured by line crossings in the light–dark plus maze. **(C)** Exposure to D-22 increased the percentage of white/total line crosses for female and male zebrafish (*p < 0.05). **(D)** D-22 treatment increased the time female zebrafish spent in white arms of the maze (*p < 0.05). Mean and S.E.M. are shown.

**Dive Tank:** There was no significant interaction [F (1,44) = 2.485, *p* = 0.1221], effect of sex [F (1,44) = 1.961, *p* = 0.1684], or effect of acute D-22 treatment [F (1,44) = 0.3468, *p* = 0.5589] on time spent in the top 2/3 of the dive tank in the follow-up study, as shown in Figure 3A.

### Light–Dark Plus Maze

The total number of line crosses or box entries did not differ by sex [F (1, 44) = 0.7577, *p* = 0.3888] or D-22 treatment [F (1, 44) = 1.381, *p* = 0.2463], and there was no significant interaction [F (1, 44) = 0.000, *p* = 0.9999], as shown in Figure 3B. However, there was a significant effect of acute D-22 treatment [F (1, 44) = 14.61, *p* < 0.0004] to increase the percentage of white/total line crosses or entries by both female [t (12) = 2.894, *p* < 0.0118] and male [t (12) = 2.511, *p* < 0.0313] zebrafish relative to their controls, as shown in Figure 3C. There was no significant interaction [F (1, 44) = 0.0730, *p* = 0.7882] or sex effect [F (1, 44) = 0.4829, *p* = 0.4908] on the percentage of white/total line crosses. Female D-22-treated zebrafish also spent significantly more time in white arms than in controls [F (1, 44) = 10.99, *p* < 0.0018, t (12) = 2.904, *p* < 0.0114], while males did not [t (12) = 1.783, *p* = 0.1563], as shown in Figure 3D. There was no interaction [F (1, 44) = 0.6288, *p* = 0.430] or sex effect [F (1,44) = 1.391, *p* = 0.2445] on the time spent in white arms.

### [^3^H] D-22 Uptake Into Zebrafish Tissues from Water

**FIGURE 4:**
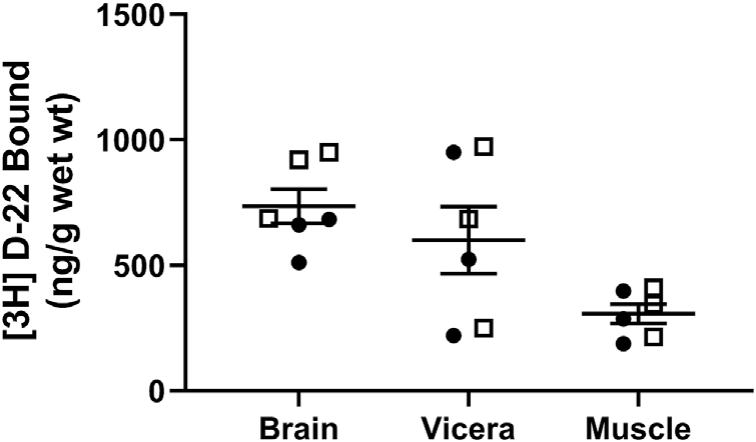
[^3^H] D-22 uptake from bath solution into zebrafish. After 10 min of *in vivo* bath exposure, zebrafish muscle had a lower D-22 content by wet weight than in either the brain or viscera. Males are shown as open boxes and females are closed circles. Mean and S.E.M. are shown.

Zebrafish exposed to 25 nM [^3^H] D-22 for 10 min took up 736 ± 68 ng/g wet weight in the brain, 601 ± 134 ng/g in the viscera, and 308 ± 38 ng/g in the muscle (*n* = 6), as shown in Figure 4. This demonstrates [^3^H] D-22 occupancy following acute bath exposure in zebrafish brain and gut are comparable and roughly double that found in muscle. This occupancy in the brain and gut is similar to zebrafish [^3^H] citalopram uptake from a 3-min bath exposure (Sackerman et al., 2010). The outcome demonstrates that [^3^H] D-22 or a metabolite that remains radiolabeled can cross the blood–brain barrier to occupy binding sites in the zebrafish brain. A prior study showed that blood–brain barrier properties in healthy adult zebrafish are comparable with those of higher mammals (Jeong et al., 2008). This finding supports the idea that bath exposures of zebrafish, paralleling systemic administration of D-22 to animals for behavioral studies is likely to occupy OCTs in the brain.

### Saturation Binding of [^3^H] D-22 in Zebrafish and Mouse Brain Homogenates

**FIGURE 5:**
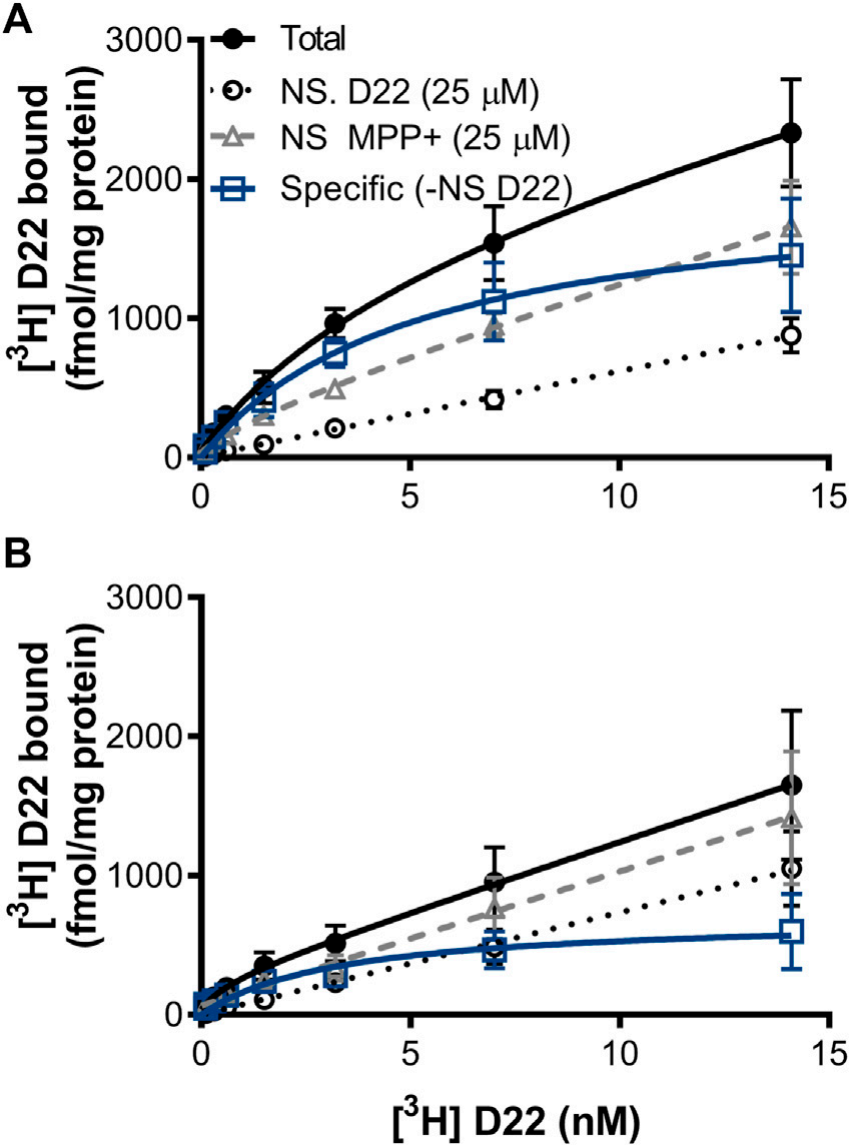
[^3^H] D-22 saturation in **(A)** zebrafish whole-brain *versus* **(B)** mouse hippocampus. A single high-affinity binding site for [^3^H] D-22 with a K_D_ of 5 ± 2.5 nM and Bmax of 1974 ± 410 fmol/mg protein was found in whole zebrafish brain, and a K_D_ of 3.3 ± 2.3 and Bmax of 704 ± 182 fmol/mg protein in mouse hippocampus, respectively, with non-specific defined by 25 μM D-22. Binding of [^3^H] D-22 was partially blocked by MPP+, *n* = 3. Mean and S.E.M. are shown.

Total binding of [^3^H] D-22 had maximal binding (Bmax) = 1,656 ± 2,839 fmol/mg protein in zebrafish whole-brain membrane homogenates pooled from females and males, and 222.7 ± 406.2 fmol/mg protein in mouse hippocampus. For total binding, the dissociation constant (K_D_) = 4.853 ± 10.59 nM in the zebrafish brain and 0.7030 ± 5.175 nM in mice. Specific [^3^H] D-22 binding, with non-specific binding defined by 25 μM D-22 plotted by linear regression and subtracted from total, had a Bmax = 1974 ± 409.7 fmol/mg protein and K_D_ = 5.172 ± 2.514 nM in zebrafish. In mice, specific [^3^H] D-22 binding had a Bmax = 704.3 ± 182.0 fmol/mg protein and K_D_ = 3.307 ± 2.308 nM. The binding of [^3^H] D-22 was partially blocked by MPP+, with a curve fit Bmax = 265.9 ± 676.1 fmol/mg protein and K_D_ = 2.124 ± 10.77 nM in zebrafish but with an ambiguous curve fit in mouse hippocampus, *n* = 3 independent experiments, as shown in Figure 5.

## Discussion

The main behavioral finding from this study is that acute exposure to D-22 at 50 mg/L increased zebrafish exploration of plus maze arms with a white background. Initially, acute bath exposure to the high-affinity mammalian OCT inhibitor D-22 reduced zebrafish box entries, the index of exploration, in the light–dark plus maze at the aforementioned dose with no other behavioral effects. For this reason, we decided not to test any higher doses of D-22 for this study. In the follow-up male vs. female experiment with the 50 mg/L D-22 dose, the percentage of white/total entries of both sexes and the amount of time spent by females in white arms was higher than those of the untreated controls (Figures 3C,D). Treatment with the mammalian stress hormone corticosterone at 25 mg/L was without effect, which may be consistent if the binding properties of zebrafish drOCT1 or drOCT2 are similar to those of mouse OCT3, as corticosterone has a lower affinity for murine OCT3 than D-22 (Koepsell et al., 2007). Our findings support the hypothesis that D-22 has anxiolytic properties relative to vehicle control treatment on zebrafish exploratory behavior in novel environments. Increased time spent in white arms after D-22 50 mg/L treatment may be indicative of an anxiolytic effect, perhaps because of reduced concern about predators from above (i.e. reduced dorsal camouflaging).

Anxiety behavior tests in zebrafish are based on predator avoidance strategies such as dwelling lower in the water column or staying on dark backgrounds (Córdova et al., 2016; Crane and Ferrari, 2017). Over the course of evolution, countershading, or dark dorsal coloration and light ventral coloration, may have been selected for in fish because it facilitates predator avoidance by background camouflage (Kelley et al., 2017). In zebrafish, countershading is under the control of melanocortin receptor 1 and a series of genes regulating agouti-signaling proteins that bind to it (Cal et al., 2019; Liang et al., 2021). Countershading is somewhat dynamic in zebrafish since within two days, fish can increase the dark pigmentation on their dorsum by the recruitment of melanosome into dorsal melanophores in response to visual exposure to a dark background or floor (Hatamoto and Shingyoji, 2008). Norepinephrine signaling, associated with the fight or flight response, is a driver of dynamic pigment shifts that zebrafish undergo to blend into their backgrounds (Xu and Xie, 2011). Taken together, it appears that substantial evolutionary pressure from predators from above may have driven zebrafish countershading and camouflaging into dark backgrounds.

By contrast, the height in the water column of the dive tank was not affected by 50 mg/L D-22. This response is akin to exploration of the middle of an open field test by rodents. Dwelling at the bottom of the tank is considered a thigmotaxic response consistent with predator avoidance. Among the experiments for this study, only the treatment with the anti-anxiety drug buspirone increased zebrafish exploration of the upper part of a dive tank, as had previously been reported to occur (Bencan et al., 2009). In other studies, drugs such as citalopram and desipramine also increase dwelling in the top 2/3 of the dive tank, while other drugs increasing time in the white arms of the plus maze, such as chlordiazepoxide, did not increase dwelling in the top of the dive tank (Sackerman et al., 2010). D-22 is therefore not the only drug with anxiolytic effects in rodents that yields different responses in the zebrafish dive tank versus plus maze.

The lack of sex-specific effects of D-22 on these behaviors is of interest, since in male zebrafish, drOCT1 and to a lesser extent drOCT2 are more highly expressed in the brain than in females (Mihaljevic et al., 2016). We observed that sex differences in mouse OCT3 (in males) vs. the plasma membrane monoamine transporter (PMAT in females) contribute more to enhanced properties of amphetamine, without knowing if differential expression of these transporters corresponds with sex in mouse brain (Clauss et al., 2021). This highlights the importance of comparing expression of uptake 2 transporters in future rodent and zebrafish studies to advance our understanding of sex-specific effects of OCTs. However, sex determination in zebrafish is a very different process than what occurs in mammals; for example, females are the heterogametic sex and chromosome 4 harbors the sex-determining gene (Aharon and Marlow, 2021). Also female zebrafish have a male-appearing phenotype if reared in warm temperatures (Hosseini et al., 2019). These kind of differences may confound efforts to effectively translate zebrafish findings to mammalian and human responses.

To complicate matters further, it appears that the mammalian expression of OCT2 and OCT3, but not OCT1, may be under epistatic control in many tissues such that only maternal coding genes are expressed (Liu et al., 2022). The control element, Antisense to Igf2r RNA Noncoding (Air), is paternally expressed and as a result, genotypes and OCT2 and OCT3 expression phenotypes in most tissues may not match in heterozygous knockout mice, since only one parental allele is active (Yotova et al., 2008). One exception to this imprinting pattern in mice may be the neurons wherein at least Igf2r was shown to be bi-allelically expressed (Yamasaki et al., 2005). Initially, maternal imprinting of mouse OCT2 and OCT3 along with insulin-like growth factor 2 receptor (Igf2R) was thought to be restricted to the mouse placenta during pregnancy (Zwart et al., 2001; Sleutels et al., 2002; Nagano et al., 2008). Recently, evidence has emerged that in mice, cattle, and in some tissue types, human OCT2 and OCT3 have mono-allelic expression (Yotova et al., 2008; Peter et al., 2017; Liu et al., 2022). Given this, future studies should consider imprinting and confirming the protein level expression of OCT2 and OCT3, and also look into the potential epistatic expression of drOCTs.

We also demonstrated with a bath exposure to [^3^H] D-22 that the drug penetrates the viscera, muscles, and brain of zebrafish in the same time frame that behavioral effects was measured. The mode of administration may affect the outcome of novel environment-based behavior tests in fish. For example, in a recent study, zebrafish were microinjected with D-22 at 0.01–10 μg/g in a salt solution vehicle, and this was found to have anxiogenic properties, increasing dark background preference, or “scotaxis” with a u-shaped curve on risk assessment based on freezing behavior (Maximino, 2021). Further studies on dosing and mode of administration that take into account the amount of D-22 reaching the brain and behavioral effects are needed to clarify its role in anxiety-related behaviors.

Characterization of high-affinity binding sites for D-22 in zebrafish or mouse brains was based on D-22 concentrations found in the brain with acute treatments that produced antidepressant-like behavioral effects in mice (Baganz et al., 2008; Horton et al., 2013). Hence the *in vitro* assays on brain membrane homogenates utilized a maximal concentration of 14 nM [^3^H] D-22, and bath exposures used 25 nM. Our goal was to capture a single high-affinity binding site most likely responsible for the beneficial behavior effects observed with 0.01–0.1 mg/kg. We did not want to use doses of D-22 approaching equivalents of 10 mg/kg because it has a sedating effect on mice (Horton et al., 2013). In the present study, 50 mg/L D-22 initially appeared to slow the exploration of the light/dark plus maze in a few fish, but did not interfere with the exploration as evidenced by box entries in the light–dark plus maze that matched controls in the follow-up study. We found that with the aid of monoamine blockers, a putative high-affinity binding site appeared to reach a specific binding plateau in the zebrafish more clearly than in the mouse saturation assays.

The pharmacological properties of zebrafish drOCT1 were assessed in concentration-dependent inhibition assays with many drugs, hormones, and xenobiotics (Mihaljević et al., 2017). D-22 was not tested in these assays for its affinity with drOCT1. *In vivo*, we now know that drOCT1 and drOCT2 and other organic cation transporters that D-22 may bind to, occur in the brain with some capacity to affect behavior after acute treatment (Mihaljevic et al., 2016). Paralleling this, although D-22 had a slightly greater affinity for human OCT3 than OCT2 or plasma membrane monoamine transporters *in vitro* (Hayer-Zillgen et al., 2002; Fraser-Spears et al., 2019), its behavioral effects on these transporters *in vivo* may involve collective inhibition of OCTs. Furthermore, D-22 may bind to other transporters or receptors which may also help mediate its behavioral effects. For example, D-22 also blocks the plasma membrane monoamine transporter (PMAT) with high affinity (Duan and Wang, 2010), and a modest affinity of D-22 for adrenergic receptors has also been reported (Russ et al., 1996). Since OCTs transport many xenobiotics, including neurotoxins and drugs, their role in exposure-induced neurodegenerative disorders could be more safely studied at high throughput in zebrafish (Gould, 2010b; Lin et al., 2021). However, binding sites in the zebrafish brain for D-22 are even less well described, so further study in this area is warranted.

## Data Availability

The raw data supporting the conclusions of this article will be made available by the authors upon reasonable request, without undue reservation.

## References

[B1] Aharon D. Marlow F. L. (2021). Sexual Determination in Zebrafish. Cell Mol Life Sci 79 (1), 8. 10.1007/s00018-021-04066-4 34936027 PMC11072476

[B2] Aoyama N. Takahashi N. Kitaichi K. Ishihara R. Saito S. Maeno N. (2006). Association between Gene Polymorphisms of SLC22A3 and Methamphetamine Use Disorder. Alcohol. Clin. Exp. Res. 30 (10), 1644–1649. 10.1111/j.1530-0277.2006.00215.x 17010131

[B3] Baganz N. L. Horton R. E. Calderon A. S. Owens W. A. Munn J. L. Watts L. T. (2008). Organic Cation Transporter 3: Keeping the Brake on Extracellular Serotonin in Serotonin-Transporter-Deficient Mice. Proc. Natl. Acad. Sci. U S A. 105 (48), 18976–18981. 10.1073/pnas.0800466105 19033200 PMC2596260

[B4] Becker M. L. Visser L. E. van Schaik R. H. Hofman A. Uitterlinden A. G. Stricker B. H. (2010). Interaction between Polymorphisms in the OCT1 and MATE1 Transporter and Metformin Response. Pharmacogenet Genomics 20 (1), 38–44. 10.1097/FPC.0b013e328333bb11 19898263

[B5] Becker M. L. Visser L. E. van Schaik R. H. Hofman A. Uitterlinden A. G. Stricker B. H. (2011). OCT1 Polymorphism Is Associated with Response and Survival Time in Anti-parkinsonian Drug Users. Neurogenetics 12 (1), 79–82. 10.1007/s10048-010-0254-5 20680652 PMC3029819

[B6] Bencan Z. Sledge D. Levin E. D. (2009). Buspirone, Chlordiazepoxide and Diazepam Effects in a Zebrafish Model of Anxiety. Pharmacol. Biochem. Behav. 94 (1), 75–80. 10.1016/j.pbb.2009.07.009 19643124 PMC2771628

[B7] Bengel D. Murphy D. L. Andrews A. M. Wichems C. H. Feltner D. Heils A. (1998). Altered Brain Serotonin Homeostasis and Locomotor Insensitivity to 3, 4-methylenedioxymethamphetamine ("Ecstasy") in Serotonin Transporter-Deficient Mice. Mol. Pharmacol. 53 (4), 649–655. 10.1124/mol.53.4.649 9547354

[B8] Bergen A. W. Javitz H. S. Krasnow R. Michel M. Nishita D. Conti D. V. (2014). Organic Cation Transporter Variation and Response to Smoking Cessation Therapies. Nicotine Tob. Res. 16 (12), 1638–1646. 10.1093/ntr/ntu161 25143296 PMC4296186

[B67] Bönisch H. (2021). Substrates and Inhibitors of Organic Cation Transporters (OCTs) and Plasma Membrane Monoamine Transporter (PMAT) and Therapeutic Implications. Handb. Exp. Pharmacol. 266, 119–167. 10.1007/164_2021_516 34495395

[B9] Bousman C. A. Glatt S. J. Everall I. P. Tsuang M. T. (2009). Genetic Association Studies of Methamphetamine Use Disorders: A Systematic Review and Synthesis. Am. J. Med. Genet. B Neuropsychiatr. Genet. 150B (8), 1025–1049. 10.1002/ajmg.b.30936 19219857

[B68] Bowman M. A. Mitchell N. C. Owens W. A. Horton R. E. Koek W. Daws L. C. (2020). Primary Lab of Origin LCD. Effect of Concurrent Organic Cation Transporter Blockade on Norepinephrine Clearance Inhibiting- and Antidepressant-Like Actions of Desipramine and Venlafaxine. Eur. J. Pharmacol. 883, 173285. 10.1016/j.ejphar.2020.173285 32697958 PMC10092728

[B10] Cal L. Suarez-Bregua P. Braasch I. Irion U. Kelsh R. Cerdá-Reverter J. M. (2019). Loss-of-function Mutations in the Melanocortin 1 Receptor Cause Disruption of Dorso-Ventral Countershading in Teleost Fish. Pigment Cel Melanoma Res 32 (6), 817–828. 10.1111/pcmr.12806 31251842

[B11] Chen E. C. Liang X. Yee S. W. Geier E. G. Stocker S. L. Chen L. (2015). Targeted Disruption of Organic Cation Transporter 3 Attenuates the Pharmacologic Response to Metformin. Mol. Pharmacol. 88 (1), 75–83. 10.1124/mol.114.096776 25920679 PMC4468641

[B12] Chen L. Takizawa M. Chen E. Schlessinger A. Segenthelar J. Choi J. H. (2010). Genetic Polymorphisms in Organic Cation Transporter 1 (OCT1) in Chinese and Japanese Populations Exhibit Altered Function. J. Pharmacol. Exp. Ther. 335 (1), 42–50. 10.1124/jpet.110.170159 20639304 PMC2957788

[B13] Clauss N. J. Koek W. Daws L. C. (2021). Role of Organic Cation Transporter 3 and Plasma Membrane Monoamine Transporter in the Rewarding Properties and Locomotor Sensitizing Effects of Amphetamine in Male andFemale Mice. Int. J. Mol. Sci. 22 (24), 13420. 10.3390/ijms222413420 34948221 PMC8708598

[B14] Connors K. A. Valenti T. W. Lawless K. Sackerman J. Onaivi E. S. Brooks B. W. (2014). Similar Anxiolytic Effects of Agonists Targeting Serotonin 5-HT1A or Cannabinoid CB Receptors on Zebrafish Behavior in Novel Environments. Aquat. Toxicol. 151, 105–113. 10.1016/j.aquatox.2013.12.005 24411165 PMC3989442

[B15] Córdova S. D. Dos Santos T. G. de Oliveira D. L. (2016). Water Column Depth and Light Intensity Modulate the Zebrafish Preference Response in the Black/white Test. Neurosci. Lett. 619, 131–136. 10.1016/j.neulet.2016.03.008 26960010

[B16] Crane A. L. Ferrari M. C. O. (2017). Patterns of Predator Neophobia: a Meta-Analytic Review. Proc. Biol. Sci. 284 (1861), 28420170583. 10.1098/rspb.2017.0583 PMC5577474 28835552

[B17] Cui M. Aras R. Christian W. V. Rappold P. M. Hatwar M. Panza J. (2009). The Organic Cation Transporter-3 Is a Pivotal Modulator of Neurodegeneration in the Nigrostriatal Dopaminergic Pathway. Proc. Natl. Acad. Sci. U S A. 106 (19), 8043–8048. 10.1073/pnas.0900358106 19416912 PMC2683105

[B18] Daws L. C. Koek W. Mitchell N. C. (2013). Revisiting Serotonin Reuptake Inhibitors and the Therapeutic Potential of "uptake-2" in Psychiatric Disorders. ACS Chem. Neurosci. 4 (1), 16–21. 10.1021/cn3001872 23336039 PMC3547475

[B19] Daws L. C. (2021). Organic Cation Transporters in Psychiatric Disorders. Handb Exp. Pharmacol. 266, 215–239. 10.1007/164_2021_473 34282486 PMC9281871

[B20] Daws L. C. (2009). Unfaithful Neurotransmitter Transporters: Focus on Serotonin Uptake and Implications for Antidepressant Efficacy. Pharmacol. Ther. 121 (1), 89–99. 10.1016/j.pharmthera.2008.10.004 19022290 PMC2739988

[B21] Duan H. Wang J. (2010). Selective Transport of Monoamine Neurotransmitters by Human Plasma Membrane Monoamine Transporter and Organic Cation Transporter 3. J. Pharmacol. Exp. Ther. 335 (3), 743–753. 10.1124/jpet.110.170142 20858707 PMC2993547

[B22] Fraser-Spears R. Krause-Heuer A. M. Basiouny M. Mayer F. P. Manishimwe R. Wyatt N. A. (2019). Comparative Analysis of Novel Decynium-22 Analogs to Inhibit Transport by the Low-Affinity, High-Capacity Monoamine Transporters, Organic Cation Transporters 2 and 3, and Plasma Membrane Monoamine Transporter. Eur. J. Pharmacol. 842, 351–364. 10.1016/j.ejphar.2018.10.028 30473490 PMC6440482

[B23] Garbarino V. R. Santos T. A. Nelson A. R. Zhang W. Q. Smolik C. M. Javors M. A. (2019). Prenatal Metformin Exposure or Organic Cation Transporter 3 Knock-Out Curbs Social Interaction Preference in Male Mice. Pharmacol. Res. 140, 21–32. 10.1016/j.phrs.2018.11.013 30423430 PMC6388691

[B24] Gasser P. J. Daws L. C. (2017). Extending the Family: Roles for Uptake2 Transporters in Regulation of Monoaminergic Signaling. J. Chem. Neuroanat. 83-84, 107–108. 10.1016/j.jchemneu.2017.07.009 28757392

[B25] Gasser P. J. (2021). Organic Cation Transporters in Brain Catecholamine Homeostasis. Handb Exp. Pharmacol. 266, 187–197. 10.1007/164_2021_470 33987762

[B69] Gould G. G. Brooks B. W. Frazer A. (2007). [(3)H] Citalopram Binding to Serotonin Transporter Sites in Minnow Brains. Basic Clin. Pharmacol. Toxicol. 101 (3), 203–210. 10.1111/j.1742-7843.2007.00100.x 17697042

[B26] Gould G. G. (2010a). “Aquatic Light/Dark Plus Maze Novel Environment for Assessing Anxious versus Exploratory Behavior in Zebrafish (*Danio rerio*) and Other Small Teleost Fish,” in Zebrafish Neurobehavioral Protocols. Editors Kalueff A.V. M Cachat J. (NY: Humana Press), 99–108. 10.1007/978-1-60761-953-6_8

[B27] Gould G. G. (2010b). “Zebrafish Biogenic Amine Transporters and Behavior in Novel Environments: Targets of Reuptake Inhibitors and Pesticide Action as Tools for Neurotoxicology Research,” in Zebrafish Models in Neurobehavioral Research. Editors Kalueff A.V. Cachat J.M. (NY: Humana Press), 181–209. 10.1007/978-1-60761-922-2_8

[B28] Gupta T. Mullins M. C. (2010). Dissection of Organs from the Adult Zebrafish. J. Vis. Exp. 4 (37), 1717. 10.3791/1717 PMC3144575 20203557

[B29] Hatamoto K. Shingyoji C. (2008). Cyclical Training Enhances the Melanophore Responses of Zebrafish to Background Colours. Pigment Cel Melanoma Res 21 (3), 397–406. 10.1111/j.1755-148X.2008.00445.x 18476910

[B30] Hayer M. Bönisch H. Brüss M. (1999). Molecular Cloning, Functional Characterization and Genomic Organization of Four Alternatively Spliced Isoforms of the Human Organic Cation Transporter 1 (hOCT1/SLC22A1). Ann. Hum. Genet. 63 (Pt 6), 473–482. 10.1017/S0003480099007770 11388889

[B31] Hayer-Zillgen M. Brüss M. Bönisch H. (2002). Expression and Pharmacological Profile of the Human Organic Cation Transporters hOCT1, hOCT2 and hOCT3. Br. J. Pharmacol. 136 (6), 829–836. 10.1038/sj.bjp.0704785 12110607 PMC1573414

[B32] Hill J. E. Makky K. Shrestha L. Hillard C. J. Gasser P. J. (2011). Natural and Synthetic Corticosteroids Inhibit Uptake 2-mediated Transport in CNS Neurons. Physiol. Behav. 104 (2), 306–311. 10.1016/j.physbeh.2010.11.012 21081135

[B33] Horton R. E. Apple D. M. Owens W. A. Baganz N. L. Cano S. Mitchell N. C. (2013). Decynium-22 Enhances SSRI-Induced Antidepressant-like Effects in Mice: Uncovering Novel Targets to Treat Depression. J. Neurosci. 33 (25), 10534–10543. 10.1523/JNEUROSCI.5687-11.2013 23785165 PMC3685842

[B34] Hosseini S. Ha N. T. Simianer H. Falker-Gieske C. Brenig B. Franke A. (2019). Genetic Mechanism Underlying Sexual Plasticity and its Association with Colour Patterning in Zebrafish (*Danio rerio*). BMC Genomics 20 (1), 341. 10.1186/s12864-019-5722-1 31060508 PMC6503382

[B35] Jeong J. Y. Kwon H. B. Ahn J. C. Kang D. Kwon S. H. Park J. A. (2008). Functional and Developmental Analysis of the Blood-Brain Barrier in Zebrafish. Brain Res. Bull. 75 (5), 619–628. 10.1016/j.brainresbull.2007.10.043 18355638

[B36] Karahoda R. Horackova H. Kastner P. Matthios A. Cerveny L. Kucera R. (2020). Serotonin Homeostasis in the Materno-Foetal Interface at Term: Role of Transporters (SERT/SLC6A4 and OCT3/SLC22A3) and Monoamine Oxidase A (MAO-A) in Uptake and Degradation of Serotonin by Human and Rat Term Placenta. Acta Physiol. (Oxf) 229 (4), e13478. 10.1111/apha.13478 32311818 PMC8345021

[B37] Kelley J. L. Taylor I. Hart N. S. Partridge J. C. (2017). Aquatic Prey Use Countershading Camouflage to Match the Visual Background. Behav. Ecol. 28 (5), 1314–1322. 10.1093/beheco/arx093

[B38] Koepsell H. Lips K. Volk C. (2007). Polyspecific Organic Cation Transporters: Structure, Function, Physiological Roles, and Biopharmaceutical Implications. Pharm. Res. 24 (7), 1227–1251. 10.1007/s11095-007-9254-z 17473959

[B39] Koepsell H. (2020). Organic Cation Transporters in Health and Disease. Pharmacol. Rev. 72 (1), 253–319. 10.1124/pr.118.015578 31852803

[B40] Krause-Heuer A. M. Fraser-Spears R. Dobrowolski J. C. Ashford M. E. Wyatt N. A. Roberts M. P. (2017). Evaluation of the Antidepressant Therapeutic Potential of Isocyanine and Pseudoisocyanine Analogues of the Organic Cation Decynium-22. Eur. J. Med. Chem. 137, 476–487. 10.1016/j.ejmech.2017.06.011 28624702 PMC5564211

[B41] Liang Y. Grauvogl M. Meyer A. Kratochwil C. F. (2021). Functional Conservation and Divergence of Color-Pattern-Related agouti Family Genes in Teleost Fishes. J. Exp. Zool B Mol. Dev. Evol. 336 (5), 443–450. 10.1002/jez.b.23041 33755299

[B42] Lin W. Yan Y. Ping S. Li P. Li D. Hu J. (2021). Metformin-Induced Epigenetic Toxicity in Zebrafish: Experimental and Molecular Dynamics Simulation Studies. Environ. Sci. Technol. 55 (3), 1672–1681. 10.1021/acs.est.0c06052 33332093

[B43] Liu X. Huo H. Jin L. Dong Y. Li D. Zhang C. (2022). Genomic Imprinting of the IGF2R/AIR Locus Is Conserved between Bovines and Mice. Theriogenology 180, 121–129. 10.1016/j.theriogenology.2021.12.013 34971973

[B44] Maier J. Niello M. Rudin D. Daws L. C. Sitte H. H. (2021). The Interaction of Organic Cation Transporters 1-3 and PMAT with Psychoactive Substances. Handb Exp. Pharmacol. 266, 199–214. 10.1007/164_2021_469 33993413

[B45] Maximino C. (2021). Decynium-22 Affects Behavior in the Zebrafish Light/dark Test. nab 3 (1), e21. 10.35430/nab.2021.e21

[B46] McMillan S. C. Géraudie J. Akimenko M. A. (2015). Pectoral Fin Breeding Tubercle Clusters: a Method to Determine Zebrafish Sex. Zebrafish 12 (1), 121–123. 10.1089/zeb.2014.1060 25548979 PMC4298160

[B47] Mihaljević I. Popović M. Žaja R. Maraković N. Šinko G. Smital T. (2017). Interaction between the Zebrafish (*Danio rerio*) Organic Cation Transporter 1 (Oct1) and Endo- and Xenobiotics. Aquat. Toxicol. 187, 18–28. 10.1016/j.aquatox.2017.03.012 28363126

[B48] Mihaljevic I. Popovic M. Zaja R. Smital T. (2016). Phylogenetic, Syntenic, and Tissue Expression Analysis of Slc22 Genes in Zebrafish (*Danio rerio*). BMC Genomics 17 (1), 626. 10.1186/s12864-016-2981-y 27519738 PMC4982206

[B49] Nagano T. Mitchell J. A. Sanz L. A. Pauler F. M. Ferguson-Smith A. C. Feil R. (2008). The Air Noncoding RNA Epigenetically Silences Transcription by Targeting G9a to Chromatin. Science 322 (5908), 1717–1720. 10.1126/science.1163802 18988810

[B70] Nies A. T. Koepsell H. Damme K. Schwab M. (2011). Organic Cation Transporters (OCTs, MATEs), *in vitro* and *in vivo* Evidence for the Importance in Drug Therapy. Handb. Exp. Pharmacol. 2011 (201), 105–167. 10.1007/978-3-642-14541-4_3 21103969

[B50] Nies A. T. Koepsell H. Winter S. Burk O. Klein K. Kerb R. (2009). Expression of Organic Cation Transporters OCT1 (SLC22A1) and OCT3 (SLC22A3) Is Affected by Genetic Factors and Cholestasis in Human Liver. Hepatology 50 (4), 1227–1240. 10.1002/hep.23103 19591196

[B51] Paull G. C. Van Look K. J. Santos E. M. Filby A. L. Gray D. M. Nash J. P. (2008). Variability in Measures of Reproductive success in Laboratory-Kept Colonies of Zebrafish and Implications for Studies Addressing Population-Level Effects of Environmental Chemicals. Aquat. Toxicol. 87 (2), 115–126. 10.1016/j.aquatox.2008.01.008 18308405

[B52] Peter B. Lancaster H. Vose C. Fares A. Schrauwen I. Huentelman M. (2017). Two Unrelated Children with Overlapping 6q25.3 Deletions, Motor Speech Disorders, and Language Delays. Am. J. Med. Genet. A. 173 (10), 2659–2669. 10.1002/ajmg.a.38385 28767196

[B53] Rappold P. M. Cui M. Chesser A. S. Tibbett J. Grima J. C. Duan L. (2011). Paraquat Neurotoxicity Is Mediated by the Dopamine Transporter and Organic Cation Transporter-3. Proc. Natl. Acad. Sci. U S A. 108 (51), 20766–20771. 10.1073/pnas.1115141108 22143804 PMC3251116

[B54] Russ H. Friedgen B. Königs B. Schumacher C. Graefe K. H. Schömig E. (1996). Pharmacokinetic and Alpha 1-adrenoceptor Antagonistic Properties of Two Cyanine-type Inhibitors of Extraneuronal Monoamine Transport. Naunyn Schmiedebergs Arch. Pharmacol. 354 (3), 268–274. 10.1007/BF00171057 8878056

[B55] Sackerman J. Donegan J. J. Cunningham C. S. Nguyen N. N. Lawless K. Long A. (2010). Zebrafish Behavior in Novel Environments: Effects of Acute Exposure to Anxiolytic Compounds and Choice of *Danio rerio* Line. Int. J. Comp. Psychol. 23 (1), 43–61. https://escholarship.org/uc/item/82h78048. 20523756 PMC2879659

[B56] Sala-Rabanal M. Li D. C. Dake G. R. Kurata H. T. Inyushin M. Skatchkov S. N. (2013). Polyamine Transport by the Polyspecific Organic Cation Transporters OCT1, OCT2, and OCT3. Mol. Pharm. 10 (4), 1450–1458. 10.1021/mp400024d 23458604 PMC3632321

[B57] Samodelov S. L. Kullak-Ublick G. A. Gai Z. Visentin M. (2020). Organic Cation Transporters in Human Physiology, Pharmacology, and Toxicology. Int. J. Mol. Sci. 21 (21), 7890. 10.3390/ijms21217890 PMC7660683 33114309

[B58] Sleutels F. Zwart R. Barlow D. P. (2002). The Non-coding Air RNA Is Required for Silencing Autosomal Imprinted Genes. Nature 415 (6873), 810–813. 10.1038/415810a 11845212

[B75] Sweet D. H. (2021). Organic cation transporter expression and function in the CNS. In Organic Cation Transporters in the Central Nervous System. Cham: Springer, 41–80. 10.1007/164_2021_463 33963461

[B59] Vialou V. Balasse L. Callebert J. Launay J. M. Giros B. Gautron S. (2008). Altered Aminergic Neurotransmission in the Brain of Organic Cation Transporter 3-deficient Mice. J. Neurochem. 106 (3), 1471–1482. 10.1111/j.1471-4159.2008.05506.x 18513366

[B60] Wultsch T. Grimberg G. Schmitt A. Painsipp E. Wetzstein H. Breitenkamp A. F. (2009). Decreased Anxiety in Mice Lacking the Organic Cation Transporter 3. J. Neural Transm. (Vienna) 116 (6), 689–697. 10.1007/s00702-009-0205-1 19280114

[B61] Xu J. Xie F. K. (2011). Α- and β-adrenoceptors of Zebrafish in Melanosome Movement: a Comparative Study between Embryo and Adult Melanophores. Biochem. Biophys. Res. Commun. 405 (2), 250–255. 10.1016/j.bbrc.2011.01.020 21219872

[B62] Yamasaki Y. Kayashima T. Soejima H. Kinoshita A. Yoshiura K. Matsumoto N. (2005). Neuron-specific Relaxation of Igf2r Imprinting Is Associated with Neuron-specific Histone Modifications and Lack of its Antisense Transcript Air. Hum. Mol. Genet. 14 (17), 2511–2520. 10.1093/hmg/ddi255 16037066

[B63] Yee S. W. Giacomini K. M. (2021). Emerging Roles of the Human Solute Carrier 22 Family. Drug Metab. Dispos. 10.1124/dmd.121.000702 PMC9488978 34921098

[B64] Yossa R. Sarker P. K. Proulx E. Saxena V. Ekker M. Vandenberg G. W. (2013). A Practical Approach for Sexing Zebrafish, *Danio rerio* . J. Appl. Aquac. 25 (2), 148–153. 10.1080/10454438.2013.792170

[B65] Yotova I. Y. Vlatkovic I. M. Pauler F. M. Warczok K. E. Ambros P. F. Oshimura M. (2008). Identification of the Human Homolog of the Imprinted Mouse Air Non-coding RNA. Genomics 92 (6), 464–473. 10.1016/j.ygeno.2008.08.004 18789384 PMC2846268

[B66] Zwart R. Sleutels F. Wutz A. Schinkel A. H. Barlow D. P. (2001). Bidirectional Action of the Igf2r Imprint Control Element on Upstream and Downstream Imprinted Genes. Genes Dev. 15 (18), 2361–2366. 10.1101/gad.206201 11562346 PMC312779

